# The Investigation of Sex Differences in the Effect of Body Mass Index

**DOI:** 10.1155/2019/1360328

**Published:** 2019-04-01

**Authors:** Xiang Xiao, Weihua Wang, Rina Sa, Lin Qiu, Feng Liu

**Affiliations:** Shaanxi Center for Disease Control and Prevention. No. 3 Jiandong Street, Beilin District, Xi'an, China

## Abstract

Although many researches regarding risk factors for hypertension have been reported, little information is known about the effect of BMI on the prevalence of hypertension considering sex differences. The aim of this study was to examine the sex difference in the prevalence of hypertension with the predicting indicator BMI. A total number of 6330 subjects in Shaanxi were examined using multivariable logistic regression to study the relationship between genders in different levels of BMI and prevalence of hypertension. Overall, females had a higher prevalence of hypertension than males, being 28.36% and 21.55%, respectively. The mean of blood pressure and the prevalence of hypertension increased as BMI getting larger. The result of multivariable logistic regression showed that obese and overweight males had higher risk of getting hypertension than their female counterparts. Further prevention of hypertension should be focused on obese and overweight males more than females and examining the mechanism of how sex differences influence the prevalence of hypertension.

## 1. Introduction

Cardiovascular disease is one of the death factors leading to the mortality worldwide as well as in China [1, 2]. The prevalence of hypertension worldwide has increased up to a severe rate according to many cardiovascular researches [3–5]. Having the largest population, a similar trend is also noted in the Chinese population over the last decades [5]. The number of estimated cases of hypertension was 59 million in 1980 and close to 100 million in 1990 in China [5]. Even though the infectious disease was well-controlled, the changes of modern lifestyle and diet have resulted in increasing the burden of chronic disease more than ever before. Due to complicity of pathogenic mechanism of hypertension, there are various risk factors, such as weight, age, body mass index (BMI) [6], and fibrinogen [7] that need to be considered when studying the risk factors contributing to morbidity.

Many researches indicated that there is a positive relationship between blood pressure and overweight [8–10]. BMI (body mass index), an index calculated by weight (in kilograms) divided by height (in meters) squared, is widely applied to define overweight and obesity in hypertension study [8]. Although a specific BMI value is carefully chosen for different populations, especially for studies aimed at Asian countries like China [9] and Japan [11], there were few studies on Asian populations such as Chinese population in terms of sex difference in the prevalence of hypertension.

The aim of this research is to discuss the effect of sex differences on BMI and the prediction of hypertension among adults aged 18 or older in rural and urban populations in China.

## 2. Materials and Method

### 2.1. Study Population

The Chronic Disease and Nutrition Surveillance Work Program 2015, conducted from Oct 2015 to Dec 2015, included the national disease surveillance points system that consisted of 302 selected disease surveillance points in all 31 provinces, autonomous regions, and municipalities in China, which guaranteed the representative sample of the general population. 6330 subjects that were over or at 18 years old were examined between Oct 2015 and Dec 2015 in Shaanxi, China, by a multistage clustering sampling method.

The first sampling level was stratified by each surveillance point including three rural locations. The second level was stratified by each rural location including two randomly sampled administrative villages. The third level was stratified by each family in rural areas including at least one neighborhood community (≥60 households). The fourth level was stratified by each community group including 45 households. At each surveillance point site, the number of inquired households must be equal to or greater than 270. The total number of inquired permanent residents (ages ≥ 18) was more important than 612. The study protocol was approved by the Ethical Review Committee of the Chinese Center for Disease Control and Prevention and other participating institutes. All study participants provided written informed consent.

### 2.2. Data Collection

Anthropometry of participants was collected in the examination center at a local station or clinics in the residential areas by investigators and local CDC staff who were trained and required to pass the test to be qualified for data collection. A standard questionnaire containing demographic characteristics, diet, lifestyle factors, and medical history was run by trained interviewers face-to-face. However, in our study, we only examined several risk factors. Only the number of cigarettes in the past 30 days was considered in terms of smoking. Education was identified as (1) uneducated, (2) primary school, (3) middle school, and (4) high school or above. Physical activity was classified into three categories including (1) work: farm work and housework, (2) communication-related activity, and (3) leisure, exercise, and sport, and housework was considered separately. Each physical activity category was divided into two levels including high and moderate based on the amount of physical efforts and the increase of breath and heart rates. In this study, only the times of moderate level of (3) leisure, exercise, and sport per week were considered. For qualitative analyses, it was divided into four categories including (1) less than 1 time per week, (2) 1-2 times per week, (3) 3-4 times per week, and (4) more than 5 times per week. Salt consumption was defined as salt intake per family members per 30 days.

Data regarding the subjects' age and sex were inquired by the questionnaire, and their weight and height were measured. The height was monitored by using a sitting height meter with subjects' shoes off. The subjects' weights were measured by TANITA HD-390 electronic scale with their casual clothes on. The cutoff of BMI is adjusted for the Department of Health in China with BMI≥24 kg/m^2^ and ≤ 28 kg/m^2^ as overweight and BMI 28 kg/m^2^ as obese [8]. Systolic blood pressure (SBP) and diastolic blood pressure (DBP) were calculated by using the automatic BP Monitor (OMRON, Model HBP-1300). Hypertension was defined as average systolic BP≥140 mm Hg, an average diastolic BP (DBP)≥90 mm Hg [8].

### 2.3. Statistical Analysis

Value was given in mean±SD if the variables are continuous and frequency are percentage if the variables are discrete. The comparison was done in Student's t-test and in *χ*^2^ respectively. Odds ratio of BMI on hypertension was obtained by multivariable logistic regression for females and males, respectively. In each logistic regression model, the dependent variables were either getting hypertension or not (“Yes”=1, “No” =0) and the independent variables were age (in years), education status (1= uneducated 2= primary school 3=middle school, 4= high school or above), smoking (number of cigarettes/ 30 days), and salt consumption (in g/ 30 days). Data was analyzed by R studio 1.1.456. Significant level was defined as p < 0.05.

## 3. Results

The characters of the study population were shown in Table 1 with gender, respectively. Mean BMI value for males was 21.74±1.74 kg/m^2^ and for females was 24.39±3.54 kg/m^2^. The prevalence of overweight and obesity was 17.38 % and 6.21 % in males and 17.65 % and 7.61 % in females. The prevalence of hypertension between two genders was in line with the prevalence of overweight, with males having a higher prevalence of hypertension than their female counterparts (27.63% > 26.94%, p< 0.05). In general, females had a lower education attainment than males especially at high school or above (7.73% < 10.24%, P < 0.05). Males constituted the majority of smoking participants whereas half of females did not smoke, and salt consumption in males was slightly higher than in females (Table 1).

The distribution of BMI was grouped by three age groups (17-45 years old, 45-68 years old, and 68 years old or above). The prevalence of overweight and obesity was 34.98% and 13.40% for males, respectively (Table 2). Females had a higher prevalence of normal and a lower overweight than males did (57.27% vs 51.62%, 29.70% vs 34.98%, Table 2). The age-specific prevalence peaked at three BMI categories in both genders at 17-45 years old.

In general, the prevalence of hypertension was higher when BMI was bigger and age was older in both genders (Table 3). The prevalence of hypertension had the highest value among the participants aged 68 years or above and BMI≥28 in females and males. As age decreases, both genders were less likely to get hypertension since the prevalence reached the minimum value in people aged 17-45 (Table 3).

Table 4 showed the odds ratio for hypertension for females and males, respectively, combined by logistic regression and adjusted for age, education status, smoking, salt consumption, and physical activity, showing the relationship between BMI and hypertension. In general, we found that overweight and obesity were risk factors for hypertension in both genders. There was a positive relationship between the BMI and the risk of getting hypertension for males and females, respectively, and combined. Males with BMI≥28 and 24-28 had higher risks of hypertension than females did. It should be noticed that males had 1.618-fold higher risk for getting hypertension than females on average. In general, overweight and obese males had a higher risk of hypertension than their female counterparts.

## 4. Discussion

Sex differences act as the risk factor in terms of hypertension as has been proved by many European studies [12, 13]. According to the study by Munter et al., men had higher risk for getting hypertension than women [14]. Our study also confirmed that obese and overweight males had a higher risk for getting hypertension than females.

Although the effect of sex on the blood pressure was not well understood, sexual hormone has been shown acting as a potential cause [15, 16]. The possible explanation is the regulation of androgens on blood pressure; however there were no clear researches stating its mechanism [17]. Dubey et al. stated that the cause of lower average blood pressure in premenopausal females is due to the ovarian hormone regulation [15]. In addition, testosterone could affect blood pressure acting as a prohypertensive hormone. Therefore, males who have higher testosterone than females are likely to have higher prevalence of hypertension [15]. Environmental and genetic factors in females and males were also suggested acting as the causes of the differences in prevalence of hypertension. In our study, the odds ratios for getting hypertension increased as the BMI value got larger in both genders, which was also concluded by Pang et al. in Chinese rural adults [10]. Similarly, the risk of getting hypertension has a positive relationship in Portugal population as well [18].

We also observed that the risk of hypertension increased largely in both males and females in the levels of BMI 24–28 and BMI≥28 (Table 4). Conversely, Futatsuka et al. stated that the risk of hypertension increased sharply in the level of BMI≥25 in female rural dwellers whereas males did not exhibit apparent increase among the different BMI groups compared with the lowest group [11]. Related to our study of a mixed population from both urban and rural areas, the influences brought by different living environments may deviate from this result. Furthermore, factors that account for modeling were different between those two studies. It would vary the odds ratios predicted by the logistic regression.

The result of multivariable logistic regression indicated that educational status and the age had a significant contribution to the odds ratios of hypertension in both genders (p < 0.05). Lloyd-Jones et al. concluded the same result; that is, the prevalence of hypertension increased with older ages [19]. Decreasing the elasticity of arterial walls and weight gain in older subjects are the possible causes leading to the increase [20]. It should not be neglected that the majority of smokers had the lowest level of BMI. This fact may to some extent contributed to the result that the factors of smoking did not have significance as the independent variable in the logistic regression model for hypertension.

In terms of the limitation of the research, cross-sectional study could potentially mask the effect of weight change on hypertension, which possibly influences the interpretation of the relationship between BMI and hypertension. In addition, alcohol consumption is associated with hypertension, [21] and we did not have adequate data for this study.

The result of sexual difference in odds ratios using BMI as predictor may be used to determine a more appropriate cutoff points of BMI when used as the indicator to predict the risk for getting hypertension of the population. Further clinic prevention of hypertension should put efforts in obese males more than females since they have higher prevalence rate of hypertension.

## 5. Conclusion

In summary, there are few researches examining the effect of body mass index on hypertension based on gender differences in Chinese population. Our research bridged the gap and found that overweight and obese males had higher prevalence rate of hypertension than females. Further prevention methods should be adapted to focus on obese and overweight males.

## Figures and Tables

**Table 1:** The main characteristics of the study population.

Variables	Male	Female	total	P value
Age*∗*(years)	52.06 ±13.91	51.00 ±13.52	51.52±13.72	0.0021
Age categories (years)				
17-45	987(14.17)	1002(15.83)	1899(30.00)	
45-68	1805(28.52)	1914(30.24)	3719(58.76)	
68 or above	381(6.02)	330(5.21)	711(11.23)	
Education status*∗*				<0.0001
(1) Uneducated %	568(8.97)	1190(18.8)	1758(27.77)	
(2) Primary school %	633(10.00)	568(8.97)	1201(30.28)	
(3) Middle school %	1234(19.49)	1000(15.80)	2234(35.29)	
(4) High school or above %	648(10.24)	489(7.73)	1137(17.96)	
Height*∗*(cm)	166.40 ± 6.60	155.69 ± 6.08	160.90 ± 8.29	<0.0001
Weight*∗*(kg)	67.15 ± 11.24	59.15 ± 9.42	63.04 ± 11.09	<0.0001
BMI*∗*(kg/m^2^)	21.74 ± 1.74	24.39 ± 3.54	24.29 ± 3.47	0.0264
Normal (BMI < 24),%	1590(51.57)	1648(50.75)	3238(51.15)	
Overweight (24≤BMI<28),%	1100(35.68)	1117(34.40)	2217(35.02)	
Obesity (BMI≥28),%	393(12.75)	482(14.84)	875(13.82)	
Hypertension %	1749(27.63)	1705(26.94)	3454(54.57)	
Smoking*∗* %				<0.0001
(1)smoker	2085(32.94)	74(1.17)	2159(34.11)	
(2)non-smoker	998(15.77)	3173(50.13)	4171(65.90)	
Salt consumption	9.70±8.11	9.64±7.19	9.67±7.65	0.7489

Mean±SD

*∗* represents statistical significance (p<0.05).

**Table 2:** Prevalence of normal, overweight, and obesity adjusted by gender and age*∗*.

	BMI<24,%	24≤BMI<28,%	BMI≥28,%	*χ*^2^	*P*
Male	51.62	34.98	13.40	102122	<0.001
17-45	58.46	57.73	59.81		
45-68	31.65	36.03	35.30		
68-97	9.89	6.24	4.89		
Female	57.27	29.70	13.03	460007	<0.001
17-45	63.98	49.64	48.52		
45-68	26.89	44.20	42.00		
68-97	9.13	6.15	9.48		
*χ*^2^	54048.1659	69159.8491	63710.9532		
P value	<0.001	<0.001	<0.001		

*∗* means weight was considered in calculation of all percentages; *χ*^2^ test.

**Table 3:** Prevalence of hypertension by age groups and BMI levels*∗*.

	BMI<24,%	24≤BMI<28,%	BMI≥28,%
Male	42.13	54.97	74.94
17-45	32.39	46.06	66.00
45-68	52.20	66.16	88.61
68-above	67.42	72.85	85.53
Female	30.70	47.59	65.82
17-45	15.46	29.08	51.74
45-68	53.05	64.53	76.80
68-above	72.14	75.28	89.22

CMH ^2^	1942375	727129	270072
P value	<0.001	<0.001	<0.001

*∗* means weight was considered in calculation of all percentages; ^2^ test.

**Table 4:** Multivariate odds ratios for risk factors grouped by sex*∗*.

	Male	Female	Both sexes
	Hypertension	Hypertension	
Sex			
male	-	-	1.000
female	-	-	1.618(1.614- 1.621)
BMI			
<24	1.000	1.000	1.000
24-28	2.010 (2.003 -2.016)	1.863 (1.858 -1.869)	1.970(1.965-1.974)
≥28	4.808 (4.786 - 4.831)	4.068 (4.046 -4.089)	4.708 (4.692-4.724)

*∗* means adjusted for age, education status, smoking, salt consumption, and physical activity; weight was considered in calculation of OR.

## Data Availability

The population data used to support the findings of this study are available from the corresponding author upon request.
